# Scientific Items

**Published:** 1884-12-20

**Authors:** 


### SCIENTIFIC ITEMS.

It is impossible, says an exchange, to form an idea of a tempest in the Polar Seas. The icebergs are like floating rocks whirled along a rapid current. The huge crystal mountains dashed against each other, backward and forward, bursting with a roar like thunder, and returning to the charge until, losing their equilibrium, they tumble over in a cloud of spray, upheaving the icefields, which fall afterwards like the crash of a whiplash on the boiling sea. The sea-gulls fly away screaming, and often a black shining whale comes for an instant, puffing, to the surface. When the midnight sun grazes the horizon, the floating mountains and the rocks are immense in a wave of beautiful purple light. The cold is by no means so insupportable as is supposed. We passed from a heated cabin, at 30° above zero, to 47° below zero in the open air, without inconvenience. A much higher degree becomes, however, insufferable, if there is a wind. At 15 degrees below zero a steam, as if from a boiling kettle, rises from the water. At once frozen by the wind, it falls in a faint powder. This phenomenon is called sea smoke. At 40° the snow and human bodies also smoke, which smoke changes at once into millions of tiny particles, like needles of ice, which fill the air, and make a light, continuous noise like the rustle of a stiff silk. At this temperature, the trunks of trees burst with a loud report, the rocks break up, and the earth opens and vomits smoking water. Knives break in cutting butter. Cigars go out in contact with the ice on the beard. To talk is fatiguing. At night the eyelids are covered with a crust of ice, which must be removed before one can open them.—*Ibid.*

A paper was read by Herr Heusner at a recent congress of German medical men, held at Magdeburg, on the effects of lightning stroke on human beings, and the author showed that when the lightning discharge passed through the skin its passage was much easier—that is, the internal organs are much more combustible than the epidermis. If this was known, it was not so well known that the brain and spinal cord are apparently conductors, and that a lightning stroke on the head does not materially injure the brain beyond shattering the nerves and causing temporary derangement. Most persons struck by lightning do not remember anything about the stroke ; but others describe a sensation such as would be caused by their being struck a heavy blow, and some have likened the shock to what they would be supposed to feel if torn into small pieces. Engineering says the subject is now happily beginning to engage the attention of physologists as well as physicians.—*Ib.*

Mr. Keely has not yet revolutionized the mechanical world or driven the builders of steam-engines and those confederated with them out of the market. He has, however, applied his motor to a breech-loading rifle, and, in a public experiment at Sandy Hook, claims to have projected twenty leaden bullets, each weighing nearly five ounces, at a velocity of over five hundred feet per second, deriving the requisite force from six drops of water and a pint of air. Not only this, but he had force enough left in his vapor tube to fire two hundred more bullets at the same rate, the tube being only just warmed up to its work when the experiment closed. He relates, with a composure which is appalling when we think of the rending and smashing he might do if he would, that he has generated a pressure of 50,000 pounds to the square inch. As for lower, every-day pressures of say 10,000 pounds to the square inch, they appear to be a mere recreation, and do not consume much valuable time, as that trifling force can be evolved in a quarter of a second. The large motor, we are told, is completed, and only needs to be adjusted. We should urge Mr. Keely to adjnst it at once, and let us know the worst; but we suppose it is better that the country should be allowed to recover from the excitement of the Presidential election before meeting this new strain upon its nervous system.—*Mechanical News.*

The process for tanning leather by electricity, a patent for which has been taken out by Mr. L. Gaulard, is thus described : The skins to be treated by this process are suspended in a solution of tannin contained in a vessel, to the center of the bottom of which is an electrode, another electrode being placed in a conduit by which this vessel communicates with another vessel. The electric current is then caused to pass in such a manner that the negative pole is in the center of the vessel, and the positive pole is in the conduit. Under these conditions, the hydrogen alone acts upon the leather, and causes the rapid destruction of the nitrogenous matter contained therein. After undergoing this treatment for a period of eight days, the liquid in the vessel is removed and replaced by a stronger solution of tannin, making about 20°. The current is then reversed, so that the pole in the middle of the vessel becomes the positive pole and the pole in the conduit the negative pole. The oxygen alone then acts upon the liquid, thereby inducing a rapid oxidization of the tannin, and its precipitation in this condition in the cells formed by the gelatine and fibrine of the hide.—*Ibid.*

The largest tree in the world is one discovered in 1874 in a grove near Tule River, in California. Though the top has been broken off, it is 240 feet high, and the diameter of the tree where it has been broken is 12 feet. This giant of the forest is called "Old Moses," from a mountain in the neighborhood, and is thought to be 4,840 years old. The hollow of trunk, which is 111 feet, will hold 150 persons, and is hung with scenes of California, is carpeted and fitted up like a drawing-room, with table, chairs, sofa, and pianoforte.—*Ibid.*

The mean temperature of the months of January and July, in London, for one hundred years is indicated as follows : The lowest mean for January occurred in 1795, and was 25.5° F. ; the highest mean for January, 47°, in 1796. The lowest mean temperature for July was 55.5°, in 1816, and the highest, 68°, in 1778 and 1859.

